# New Imaging Strategies Using a Motion-Resistant Liver Sequence in Uncooperative Patients

**DOI:** 10.1155/2014/142658

**Published:** 2014-08-27

**Authors:** Bong Soo Kim, Kyung Ryeol Lee, Myeng Ju Goh

**Affiliations:** Department of Radiology, Jeju National University Hospital, Jeju National University School of Medicine, 1753-3 Ara-1-dong, Jeju-si, Jeju-do 690-716, Republic of Korea

## Abstract

MR imaging has unique benefits for evaluating the liver because of its high-resolution capability and ability to permit detailed assessment of anatomic lesions. In uncooperative patients, motion artifacts can impair the image quality and lead to the loss of diagnostic information. In this setting, the recent advances in motion-resistant liver MR techniques, including faster imaging protocols (e.g., dual-echo magnetization-prepared rapid-acquisition gradient echo (MP-RAGE), view-sharing technique), the data under-sampling (e.g., gradient recalled echo (GRE) with controlled aliasing in parallel imaging results in higher acceleration (CAIPIRINHA), single-shot echo-train spin-echo (SS-ETSE)), and motion-artifact minimization method (e.g., radial GRE with/without k-space-weighted image contrast (KWIC)), can provide consistent, artifact-free images with adequate image quality and can lead to promising diagnostic performance. Understanding of the different motion-resistant options allows radiologists to adopt the most appropriate technique for their clinical practice and thereby significantly improve patient care.

## 1. Introduction

Magnetic resonance (MR) imaging is an excellent imaging modality to evaluate the liver which is vulnerable to a spectrum of neoplastic and nonneoplastic conditions, and MR imaging can provide various types of information that it is able to generate in order to demonstrate reliable display of disease process. Artifacts produced by physiological motion caused by patient respiration and bowel peristalsis are a challenge when using MR imaging for hepatic imaging. Motion artifacts may also distinctly degrade the quality of MR images. Therefore, suppression of motion artifacts is the prime determinant of the diagnostic efficacy of liver MR imaging. With the recent advances in the development of high-performance gradient coils and phased-array torso coils as well as the continuing evolution of software, new pulse sequences have become available for motion-resistant liver MR imaging. In this paper, we discuss motion-resistant MR imaging in terms of its technical basis, advantages and limitations, and primary clinical applications.

## 2. MR Imaging Sequences

Motion-resistant protocols achieve improved image quality in the setting of noncooperative patients. A strategy for achieving optimal images includes temporally matching the data acquisition and short scanning time by acquiring the critical data for image creation during a short breath-hold time and thus rendering this technique relatively insensitive to patient motion [1]. Another approach used to minimize the impact of respiratory motion in the liver is to modify the data acquisition so as to minimize effects of motion using a radial k-space sampling scheme (Table 1) [2].

### 2.1. T1-Weighted Sequences

#### 2.1.1. Magnetization-Prepared Rapid-Acquisition Gradient Echo (MP-RAGE) Sequence

**Figure 1:**
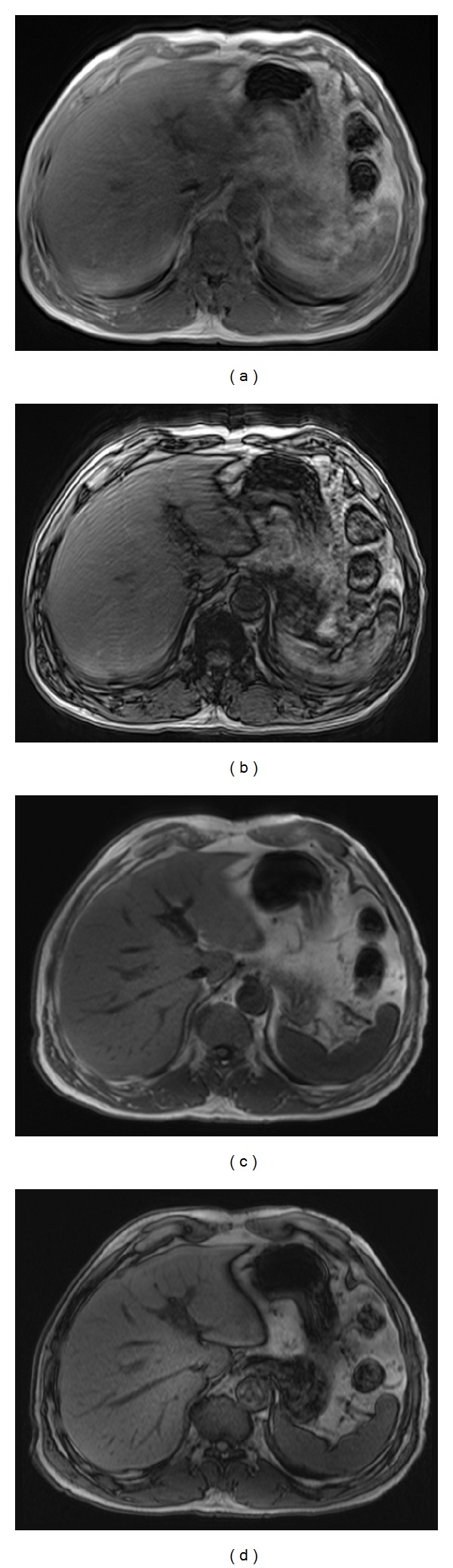
MR images obtained in a 67-year-old man. Standard T1-weighted 2D SGE in-phase (a) and out-of-phase (b) images show severe motion artifacts caused by his breathing. The motion-resistant protocol using axial T1-weighted 2D MP-RAGE in-phase (c) and T1-weighted 2D MP-RAGE out-of-phase (d) images demonstrate substantially reduced artifacts and motion-free, high-quality images in this patient.

The dual GE in-phase (IP) and opposed-phase (OP) sequence has become a routine component of liver MR imaging. This sequence is useful for detecting the presence of fat within the liver and lipid within adrenal masses in order to characterize them as adenomas [1, 3, 4]. Dual GE IP/OP imaging is performed as multiline GE acquisitions that require patients to suspend their respiration as the sequences are generally 10–20 seconds in duration. A new application of MP-RAGE IP/OP images is able to substitute standard dual GR IP/OP imaging with moderate image quality in the elderly, severely debilitated, and in young children (Figure 1) [5–7]. MP-RAGE operates as a slice-by-slice, single-shot technique used during free breathing. This sequence can be used to obtain motion-free images with acquisition times as short as one second. Magnetization preparation is currently performed with a 180° inversion pulse in order to impart greater T1-weighted information. The sequence acquires data using a very short repetition time (TR) and low flip-angle excitation pulses in order to reduce the acquisition time and maintain the prepared magnetization. SGE IP/OP technique acquires dual echo images simultaneously which has no spatial misregistration between slices, and MP-RAGE IP/OP imaging is a single echo technique, acquired in two separate acquisitions. Therefore, respiratory triggering technique could be needed for preventing misregistration, which results in long acquisition time. Another disadvantage of MR-RAGE IP/OP imaging is presence of bounce-point artifacts seen on inversion recovery sequence. They are seen at the interface between hepatic vessels and hepatic parenchyma. Notwithstanding these disadvantages, only MP-RAGE IP/OP sequence would be applicable for acquisition of chemical shift imaging in an usual clinical setting, when 2D-GRE is not feasible or might generate nondiagnostic images, such as in the case of uncooperative patients and pediatrics [5].

### 2.2. T2-Weighted Sequences

#### 2.2.1. Single-Shot, Echo-Train, and Spin-Echo (SS-ETSE) Sequence

**Figure 2:**
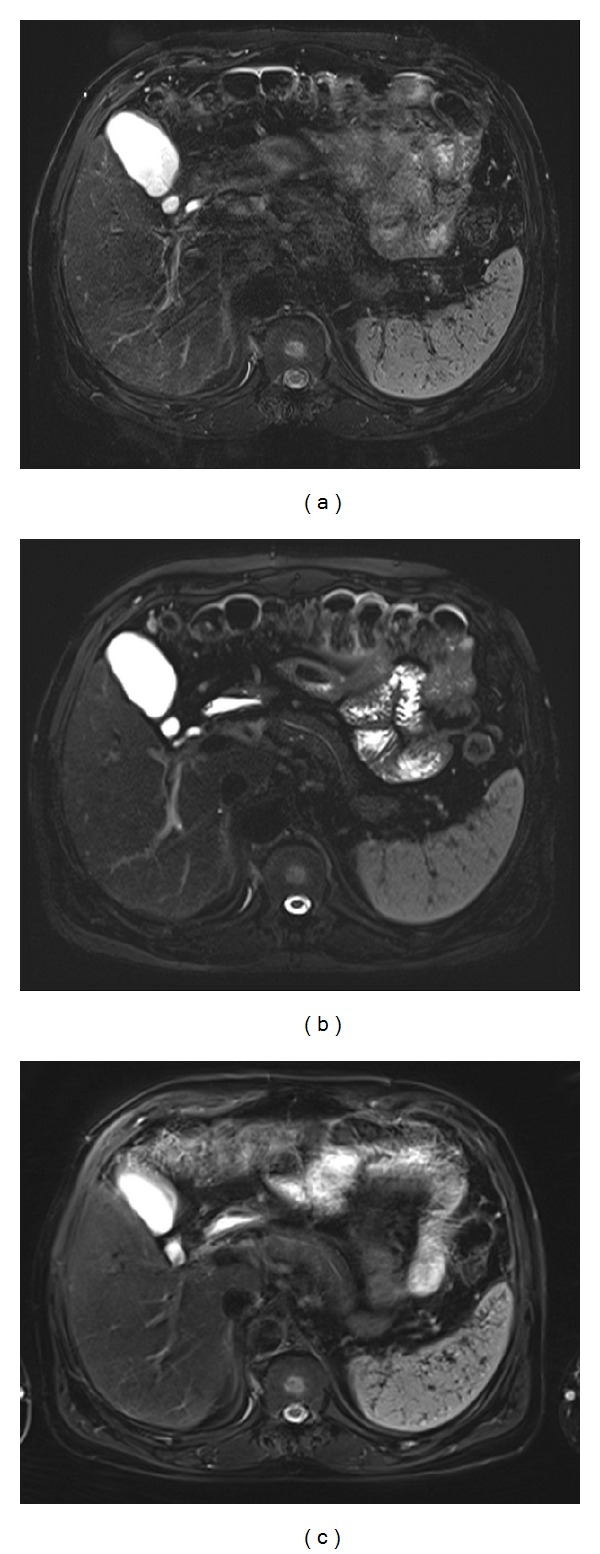
MR images obtained in a 53-year-old man with chronic liver disease. The conventional T2-weighted turbo spin-echo image (a) shows motion artifacts in the right posterior section of the liver. The artifact is improved on T2-weighted SS-ETSE (b) and BLADE (c) (proprietary name for periodically rotated overlapping parallel lines with enhanced reconstruction (PROPLLER) in MR systems from Siemens Medical Solutions) MR images. Much better sharpness of the liver edge and pancreas and clearer depiction of the main pancreatic duct are seen on T2-weighted SS-ETSE (b) and BLADE (c).

The SS-ETSE sequence is the most widely available, fastest imaging method. This technique is used to fill the entire k-space after a single excitation. It measures only half of the k-space lines and uses the k-space symmetry to regenerate the other half [2]. Images are obtained in less time with one second and with virtually no motion artifact even during free breathing [8]. The SS-ETSE sequence is the mainstay tool for water-sensitive imaging of the upper abdomen in noncooperative patients (Figure 2). In cases of extreme motion, multiple repeated imaging loops of SS-ETSE can offer the benefit of an increased signal-to-noise ratio (SNR) and resolution in conjunction with a motion-correction algorithm, snapshot-to-volume reconstruction [9]. This technique provides low contrast as there is a relatively small T2 difference between diseased and normal tissue and it is routinely coupled with fat suppression in order to increase its sensitivity to detect hepatic lesions.

#### 2.2.2. Periodically Rotated Overlapping Parallel Lines with Enhancement Reconstruction (PROPELLER)

This T2-weighted radial imaging technique, introduced by Pipe [10], is intrinsically self-navigated as oversampling of the central k-space can be used to reduce motion characteristics and to correct motion artifacts. The motion artifacts in this technique are not propagated in the phase-encoding direction as that changes with each radial section (Figure 2). Instead, the movement artifacts are dispersed throughout the radial sections (streak artifact) and become less prominent. The disadvantages of radial k-space filling include its longer imaging time and residual artifacts for through-plane motion as this technique is section-selective and useful for decreasing in-plane motion artifacts [2].

### 2.3. Contrast-Enhanced Fat-Suppressed T1-Weighted Three-Dimensional (3D) Gradient Recalled Echo (GRE) Sequence

Contrast-enhanced dynamic MR imaging has a key role in the detection and characterization of focal liver lesions when using a nonspecific extracellular GBCA [8, 11]. The hepatic arterial-dominant phase is most useful for detecting hypervascular liver tumors such as hepatocellular carcinomas and hypervascular metastases. Hepatic arterial phase images can be more frequently degraded than images from other phases, for example, early hepatic venous phase or interstitial phase. On dynamic MR imaging, poor image quality is generally caused by respiratory motion artifacts. Therefore, strategies to compensate for patient motion must be developed for uncooperative patients. Two motion-resistant methods achieve improved hepatic arterial-dominant phase in uncooperative patients. First, the radial GRE Sequence can be used to modifying the data acquisition in order to minimize the motion effects. Second, the images are acquired during a short breath-hold time before deterioration resulting from respiration using the newly developed, parallel acceleration technique (CAIPIRINHA technique).

#### 2.3.1. Motion-Resistant, Free-Breathing, Three-Dimensional (3D) Radial Fat-Suppressed GRE Sequence

**Figure 3:**
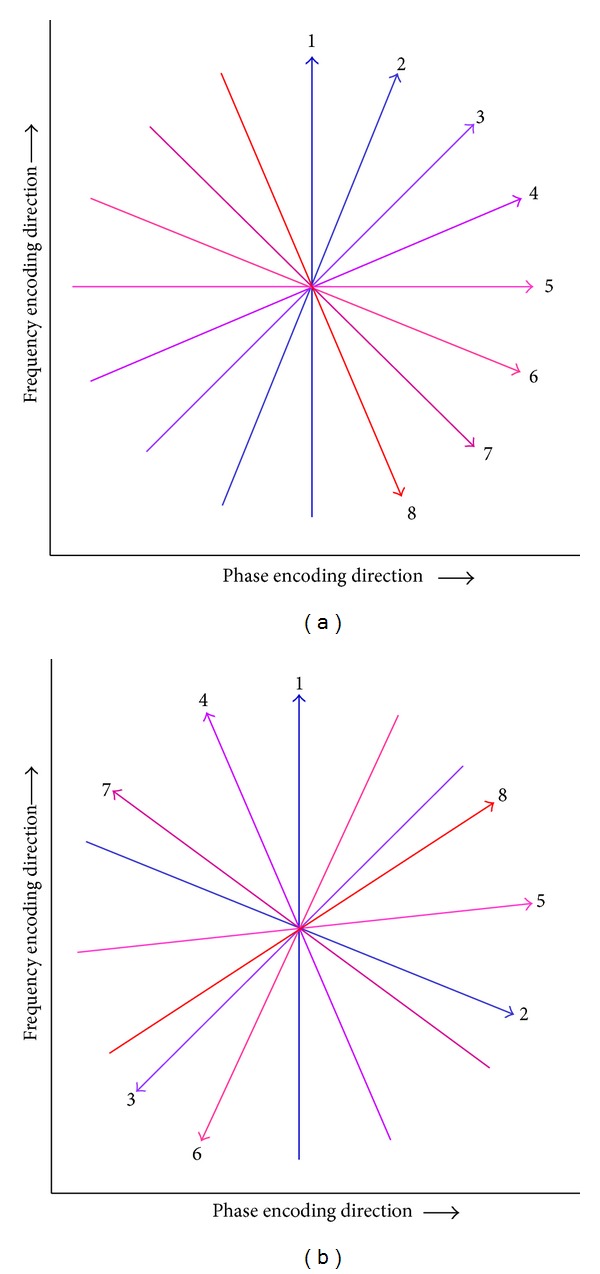
Radial k-space sampling system on the interleaved angle-bisection and golden-angle acquisitions. Radial spokes on the interleaved angle-bisection are acquired at a short angular distance. In golden-angle acquisition, the angle is continuously increased by a large angular distance (111.25°), which leads to uniform coverage for an arbitrary number of spokes.

**Figure 4:**
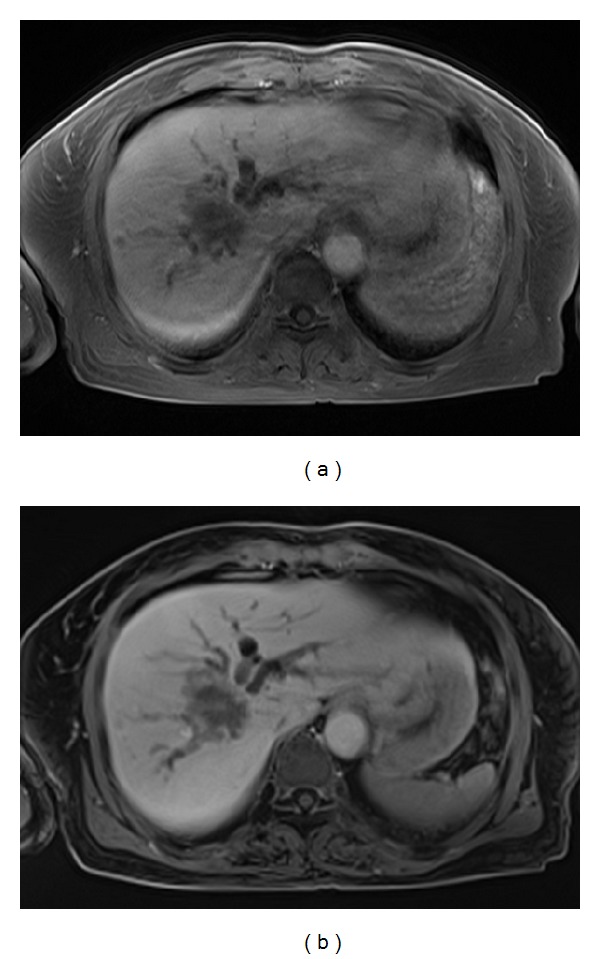
MR images obtained in a 70-year-old woman with an intrahepatic mass-forming cholangiocarcinoma. The T1-weighted 3D conventional breath-hold GRE image (a) shows blurred tumor resolution and dilated intrahepatic bile ducts caused by moderate motion artifacts. The free-breathing T1-weighted 3D radial GRE image (b) shows clear definition of the liver tumor and renders increasing conspicuity of bile-duct invasion. No motion artifacts are present in this image.

The 3D T1-weighted volumetric-interpolated GRE sequence with rectilinear Cartesian k-space sampling during single breath-hold, as usually performed, is sensitive to motion artifacts and can result in suboptimal images in patients who cannot adequately hold their breath. Recently, a more motion-robust, 3D GRE sequence has been developed (3D Radial GRE) that uses the “stack-of-stars” scheme to acquire data in a radial, spoke-wheel fashion [12–19]. 3D radial GRE has a higher sampling density at central k-space and under-samples at the k-space margins, with the net effect that images are less sensitive to motion artifacts caused by phase errors [14, 16–18]. There are two radial acquisition methods of the interleaved, angle-bisection scheme and the golden-angle scheme, which differ primarily in the temporal order of radial samplings [19]. In the interleaved angle-bisection scheme, radial spokes are acquired at a small, angular distance, whereas golden-angle acquisition acquires each spoke at a large angular distance from the preceding spoke (Figure 3). The value of golden-angle sampling is that it provides more uniform angular coverage of the k-space than interleaved, angle-bisection sampling. The 3D radial GRE sequence can provide excellent motion-controlled images with high spatial resolution in noncooperative patients, especially sedated children, at the expense of a longer acquisition time (approximately 2-3 minutes) (Figure 4) [12]. Residual artifacts caused by motion and flow are spread in two dimensions and appear as streaking artifacts on radial imaging, rather than the ghost-like artifacts seen in Cartesian phase-encoding acquisition MR imaging.

#### 2.3.2. Contrast-Enhanced, Motion-Resistant, Dynamic 3D Radial GRE Using k-Space Weighted Image Contrast (KWIC)

**Figure 5:**
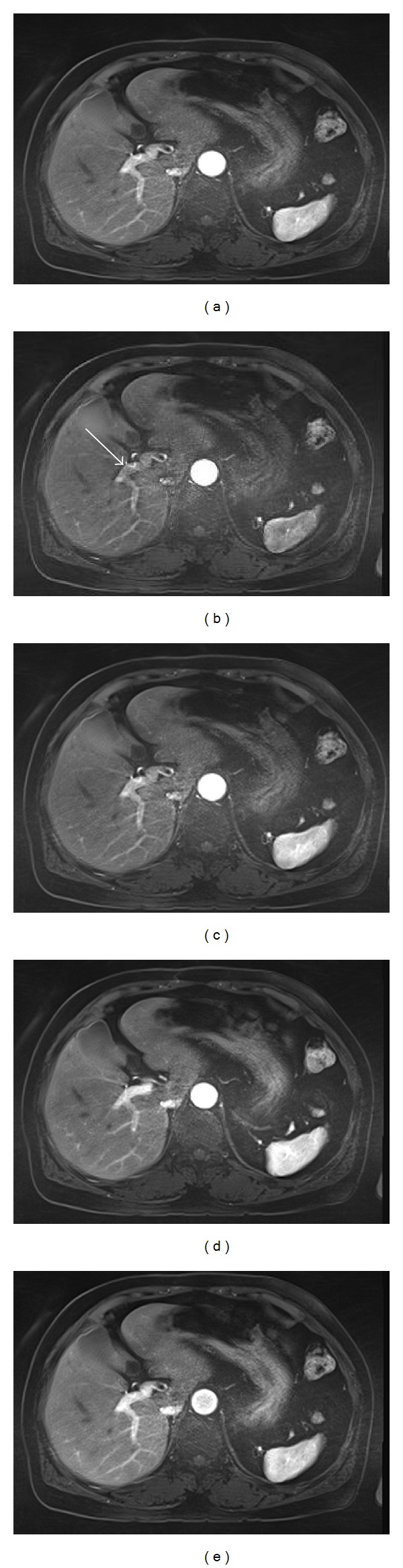
MR images obtained in a 68-year-old woman for workup of metastasis. Contrast-enhanced dynamic 3D radial GRE using KWIC during hepatic arterial dominant phase imaging comprised four subframe images. The full-frame 3D radial GRE (a) image shows homogenous enhancement in the portal vein, but not in the hepatic vein. The first subframe image (b) obtained at 5-second temporal resolution demonstrates minimal portal venous enhancement with laminar flow (arrow). On the next serial three subframe images ((c)–(e)), the portal vein and hepatic parenchyma have gradually increasing enhancement.

KWIC which is a recently proposed image reconstruction method provides time-resolved sub-frame images as well as full-frame images. The 3D radial KWIC sequence may be able to provide useful motion-insensitive data for dynamic MR imaging, that is, time-resolved subframe images, with reasonably high temporal (approximately 3–5 seconds) and spatial resolution (Figure 5) [15, 19–21]. This reconstruction technique can produce four to 16 subframe KWIC images per full-frame gridded image. Recently, Chandarana et al. reported that free-breathing, dynamic, 3D radial GRE using golden-angle sampling and compressed sensing allowed high image quality in the hepatic arterial and portal venous phases which were obtained during continuous scanning of 90 seconds, comparable to the 3D, breath-hold GRE sequence [19].

#### 2.3.3. 3D GRE with Controlled Aliasing in Parallel Imaging Results in Higher Acceleration (CAIPIRINHA) Technique

**Figure 6:**
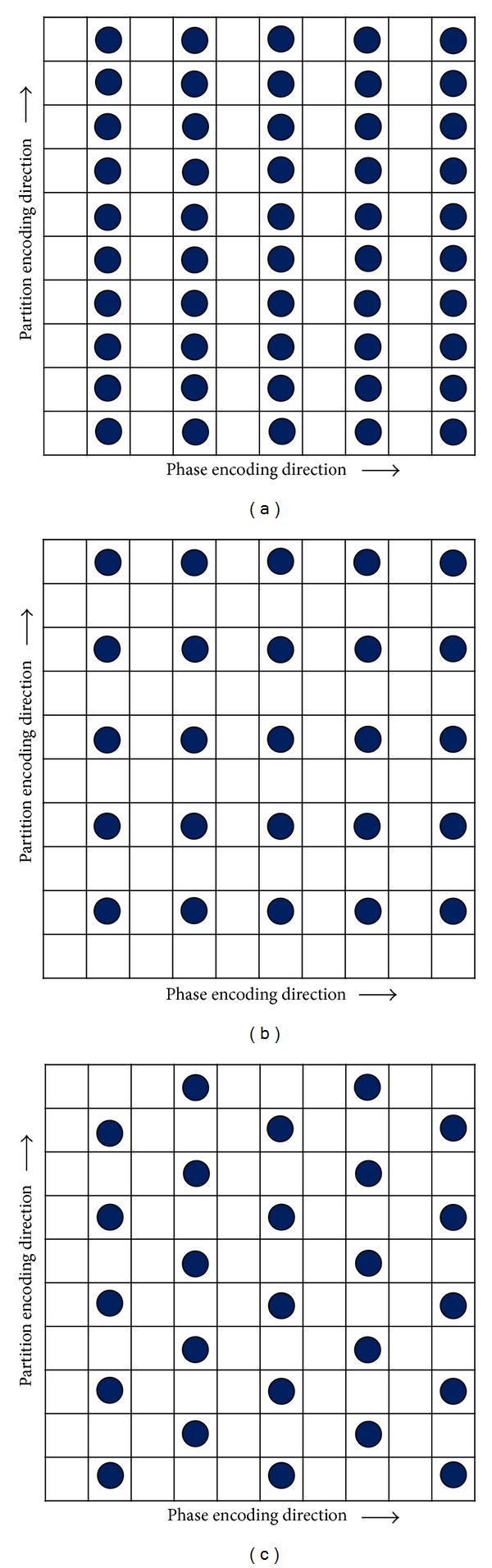
Concept of the under-sampling pattern of the CAIPIRINHA technique. The lattice work represents k-space. The black dots indicate the sampling positions, and the empty cells imply omitted sampling. (a) In the 1D parallel acquisition technique for a given total acceleration factor of 2, the under-sampling pattern can occur only in the phase-encoding direction. (b) In the 2D standard parallel acquisition technique without delta shift for a given total acceleration factor 4 (2 × 2), the under-sampling pattern can be generated concurrently in the phase- and partition-encoding directions. (c) In the 2D CAIPIRINHA technique with a delta shift of 1 for a given total acceleration factor 4 (2 × 2), data sampling is acquired by shifting every other filled partition-encoding line by as much as 1 in the partition-encoding direction. By shifting the sampling positions in a well-directed manner, aliasing can be shifted and reduced.

**Figure 7:**
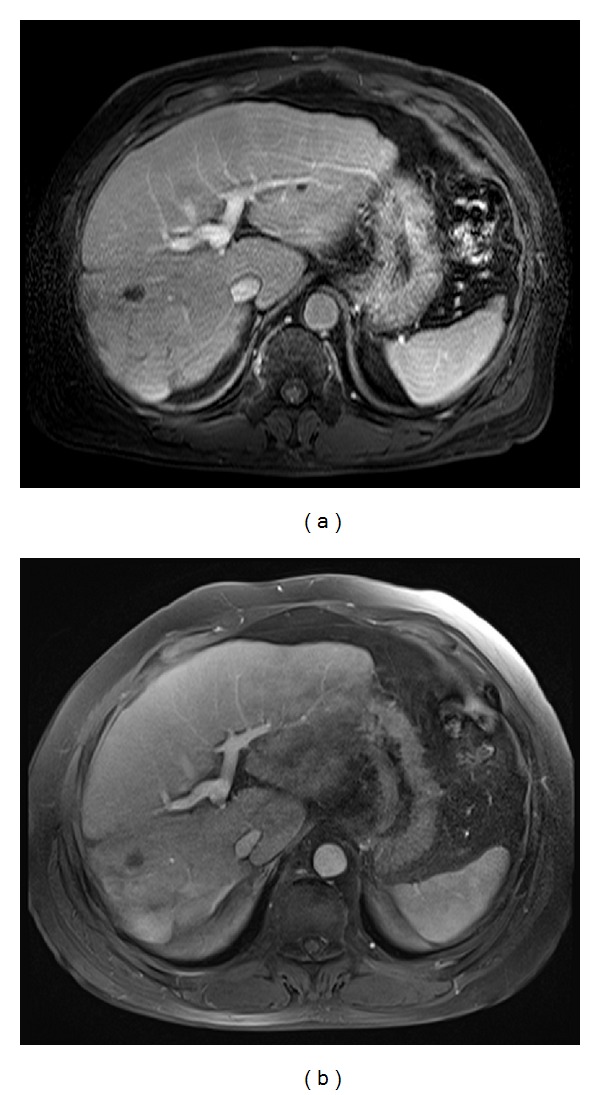
MR images in a 65-year-old man with liver cirrhosis. The conventional breath-hold T1-weighted 3D GRE sequence (acquisition time: 23 seconds) and the CAIPIRINHA 3D GRE sequence (acquisition time: 12 seconds) were obtained during the early hepatic venous phase. T1-weighted 3D GRE (a) shows blurred resolution of the intrahepatic vessel and the hepatic margin caused by motion artifacts. Much greater sharpness of the hepatic edge and a clearer depiction of the intrahepatic vessel are shown on the T1-weighted 3D CAIPIRINHA GRE sequence.

Parallel imaging reduces the imaging time by under-sampling in the phase-encoding direction according to an acceleration factor (*R*). The limitation of parallel imaging reconstructions are a decrease in the signal-to-noise ratio (SNR) that heavily depends on the geometry of the coil array used and residual aliasing artifacts at high acceleration factors, which lead to a loss in image quality [22]. With the CAIPIRINHA technique, the acquisition pattern is modified in a controlled way by shifting the sampling positions from their normal positions with respect to each other in the partition-encoding direction, which is often referred to as the delta shift [23, 24]. This reduces the quantity or severity of aliased pixels, which leads to a more innovative parallel imaging reconstruction with fewer residual aliasing artifacts (Figure 6). This technique could provide higher quality images even at high acceleration factors, which is especially attractive for use in patients with breath-hold difficulties or those with poor compliance (Figure 7).

**Figure 8:**
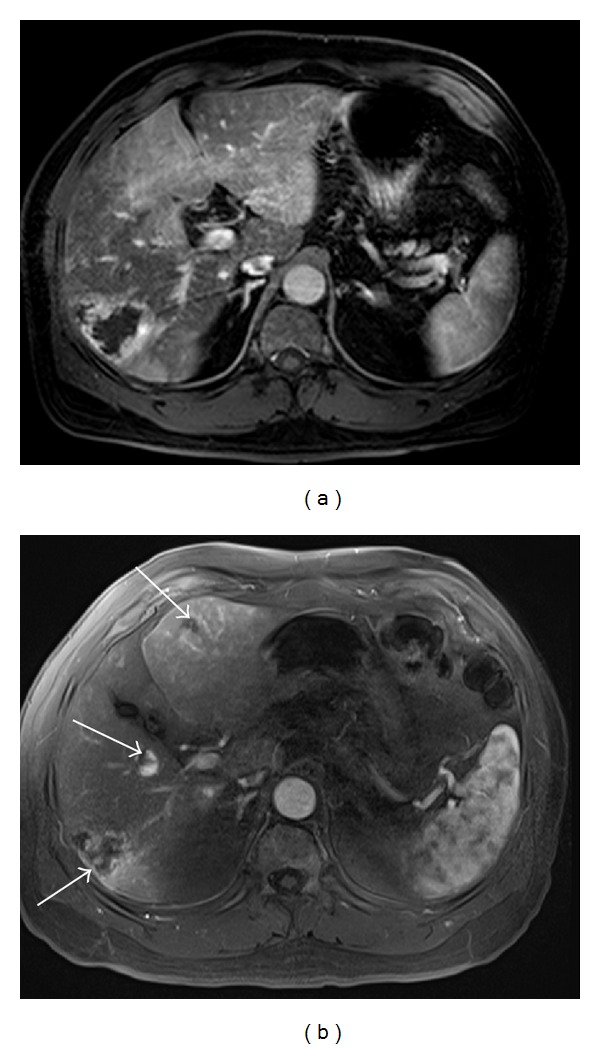
MR images obtained in a 61-year-old man with multiple hemangiomas. (a) A hepatic arterial dominant phase image using conventional 3D GRE during a 20-second breath-hold shows respiratory motion artifacts. (b) A hepatic arterial dominant phase image using the CAIPIRINHA 3D GRE sequence during a 12-second breath-hold demonstrates good image quality without artifacts, high spatial resolution, and a well-timed late arterial image and, thus, resulting in detecting an increasing number of hemangiomas (arrows).

**Figure 9:**
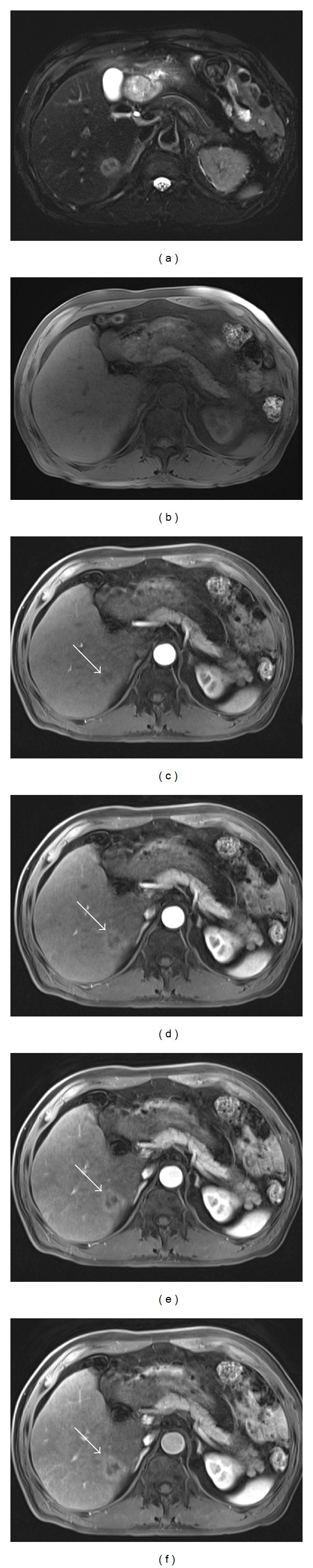
MR images obtained in 63-year-old man with a hepatocellular carcinoma. A hepatocellular carcinoma in segment 6 is clearly shown on T2-weighted fat-suppressed SS-ETSE (a). Precontrast (b) and gadoxetic acid- (Gd-EOB-DTPA-) enhanced 3D CAIPIRINHA GRE obtained using view-sharing technique ((c)–(f)) images during multiple hepatic arterial dominant phases at 4-second temporal resolution, show gradually increasing hypervascularity of the hepatocellular carcinoma (arrows) compared with that of the liver parenchyma.

The CAIPIRINHA technique can reduce the overall acquisition time from 20 to 10 seconds and can shorten the breath-holding time needed to obtain hepatic arterial-dominant phase images (Figure 8) [25]. 3D GRE with CAIPIRINHA technique have better resolution than radial GRE. Therefore, dynamic liver MR imaging using CAIPIRINHA technique is preferentially attempted in patients who can suspend breath-hold of approximately 10 seconds. The primary intention of using dynamic liver MR imaging using CAIPIRINHA technique is judged by the scanning technologist who made determination based on tolerability by patients after performing precontrast scanning. Recently, the rate of transient severe motion (TSM) during the arterial phase has been more frequently reported on dynamic MR imaging using gadoxetate disodium (17% (17 of 99 patients)) [26] which has been increasingly used for the detection and characterization of focal lesions [27, 28]. Pietryga et al. reported that rapid, triple, arterial-phase imaging using the CAIPIRINHA technique during a single breath-hold is an effective method for obtaining well-timed, late arterial-phase image sets with reduced motion artifact in most patients who experience TSM in the arterial phase [29]. A recently proposed method that includes time-resolved imaging using the echo-sharing technique, such as time-resolved imaging with interleaved stochastic trajectories (TWIST; Siemens) or time-resolved imaging of contrast kinetics (TRICKS; GE), may also provide motion-resistant imaging and have higher signal-to-noise ratios [30, 31]. The CAIPIRINHA technique combined with echo-sharing has a higher sampling density for the central k-space and under-samples for the k-space periphery. This technique can allow whole-liver coverage with multiple, arterial-phase acquisitions (4–15 sets) of higher temporal resolution (approximately 2–5 seconds) during one breath-hold (Figure 9) [31]. The multiple, arterial acquisitions provide the benefit of robustness to some artifacts, in which artifacts that compromise one data set, if transient, may not disturb others and provide more detailed enhancement characteristics of hepatic lesions [29, 32, 33].

## 3. Conclusion

Liver MR imaging using a variety of motion-resistant sequences is able to enhance the quality of liver imaging in patients who cannot remain motionless. In uncooperative patients, the combination of faster imaging, data under-sampling and the motion-artifact minimization method may eventually provide spatial resolution and image quality that equals that available using conventional MR sequences in cooperative patients. With the continuing improvements in sequence technology and with more clinical experience, the utility of motion-resistant MR imaging for evaluating the liver in uncooperative patients will be validated and will improve.

## Figures and Tables

**Table 1:** Parameters for the motion-resistant protocol used for 3.0T MR imaging scanners.

Parameter	Precontrast sequences			Postcontrast sequences			
	T1-weighted 2D MP-RAGE in-phase/out-of-phase	T2-weighted SS-ETSE	BLADE	T1-weighted 3D-GRE with CAIPIRINHA	T1-weighted 3D-GRE with CAIPIRINHA (echo-sharing technique)	T1-weighted radial 3D-GRE	T1-weighted radial 3D-GRE (KWIC technique)
Plane of acquisition	Axial	Axial, coronal	Axial	Axial, coronal	Axial	Axial	Axial
TR (milliseconds)	1500	1200	3910	4.6	4.7	3.4	3.1
TE (milliseconds)	2.3/3.4	150	105	2.2	1.5	1.7	1.5
TI (milliseconds)	1200						
Flip angle (°)	20	120	160	12	12	12	12.5
Echo train length		102	23	1	2		
Parallel imaging	GRAPPA 2	GRAPPA 2	GRAPPA 2	2D CAIPIRINHA 2 × 2	2D CAIPIRINHA 2 × 2	GRAPPA 2	GRAPPA 2
BW/pixel (Hz)	210/180	710	300	395	870	870	1040
Matrix (frequency × phase)	256 × 173	320 × 213	320 × 320	384 × 227	384 × 211	384 × 384	320 × 320
FOV (mm)	302 × 304	302 × 394	380 × 380	302 × 227	296 × 299	340 × 340	340 × 340
Number of sections	36	36	36	80	80	80	80
Section thickness (mm)	5	5	5	2.5	2.5	2.5	3.0
Intersectional gap (mm)	1.5	1.5	1.5	0	0	0	0
Number of signal acquisitions	1	1		1	1	1	1
Fat suppression	None	Fat sat for axial, none for coronal	Fat sat	Fat sat	Fat sat	Fat sat	Fat sat
Respiratory control	BI	BI	FB	BH	BH	FB	BH

BH, breath-hold; BI, breathing-independent; BLADE, proprietary name for periodically rotated overlapping parallel lines with enhanced reconstruction (PROPELLER) in MR systems from Siemens Medical Solutions; BW, bandwidth; Hz, Hertz; CAIPIRINHA, controlled aliasing in parallel imaging results in higher acceleration; FB, free breathing; FOV, field of view; GRE, gradient recalled echo; KWIC, k-space weighted image contrast; MP-RAGE, magnetization-prepared rapid-acquisition gradient echo; sat, saturation; SS-ETSE, single-shot echo-train spin-echo; TE, echo time; TI, inversion time; TR, repetition time.
